# Core Health Outcomes In Childhood Epilepsy (CHOICE): protocol for the selection of a core outcome set

**DOI:** 10.1186/s13063-017-2323-7

**Published:** 2017-11-28

**Authors:** Christopher Morris, Colin Dunkley, Frances M. Gibbon, Janet Currier, Deborah Roberts, Morwenna Rogers, Holly Crudgington, Lucy Bray, Bernie Carter, Dyfrig Hughes, Catrin Tudur Smith, Paula R. Williamson, Paul Gringras, Deb K. Pal

**Affiliations:** 1 Peninsula Cerebra Research Unit (PenCRU) & NIHR Collaboration for Leadership in Applied Health Research and Care South West Peninsula (PenCLAHRC), University of Exeter Medical School, Exeter, UK; 2 Sherwood Forest Hospitals NHS Foundation Trust, Sutton in Ashfield, UK; 3 Noah’s Ark Children’s Hospital for Wales, Cardiff and Vale University Health Board, Cardiff, UK; 4 Institute of Psychiatry, Psychology & Neuroscience, London, UK; 5 Evidence Synthesis Team, NIHR Collaboration for Leadership in Applied Health Research and Care South West Peninsula (PenCLAHRC), University of Exeter Medical School, Exeter, UK; 6 Institute of Psychiatry, Psychology and Neuroscience, King’s College London, London, UK; 7 Children, Young People and Families, Edge Hill University, Ormskirk, UK; 8 Edge Hill University, Ormskirk, UK; 9 Centre for Health Economics and Medicines Evaluation, Bangor University, Bangor, UK; 10 MRC North West Hub for Trials Methodology Research, Department of Biostatistics, University of Liverpool, Liverpool, UK; 11 Evelina London Children’s Hospital and King’s College London, London, UK; 12 Institute of Psychiatry, Psychology & Neuroscience, King’s College London, London, UK

**Keywords:** Epilepsy, Children, Young people, Paediatric, Core outcome set

## Abstract

**Background:**

There is increasing recognition that establishing a core set of outcomes to be evaluated and reported in trials of interventions for particular conditions will improve the usefulness of health research. There is no established core outcome set for childhood epilepsy. The aim of this work is to select a core outcome set to be used in evaluative research of interventions for children with rolandic epilepsy, as an exemplar of common childhood epilepsy syndromes.

**Methods:**

First we will identify what outcomes should be measured; then we will decide how to measure those outcomes. We will engage relevant UK charities and health professional societies as partners, and convene advisory panels for young people with epilepsy and parents of children with epilepsy. We will identify candidate outcomes from a search for trials of interventions for childhood epilepsy, statutory guidance and consultation with our advisory panels.

Families, charities and health, education and neuropsychology professionals will be invited to participate in a Delphi survey following recommended practices in the development of core outcome sets. Participants will be able to recommend additional outcome domains. Over three rounds of Delphi survey participants will rate the importance of candidate outcome domains and state the rationale for their decisions. Over the three rounds we will seek consensus across and between families and health professionals on the more important outcomes. A face-to-face meeting will be convened to ratify the core outcome set. We will then review and recommend ways to measure the shortlisted outcomes using clinical assessment and/or patient-reported outcome measures.

**Discussion:**

Our methodology is a proportionate and pragmatic approach to expediently produce a core outcome set for evaluative research of interventions aiming to improve the health of children with epilepsy. A number of decisions have to be made when designing a study to develop a core outcome set including defining the scope, choosing which stakeholders to engage, most effective ways to elicit their views, especially children and a potential role for qualitative research.

## Background

Epilepsy is an umbrella term covering a number of conditions including around 30 different syndromes defined by persisting tendency for seizures. Our focus is on school-aged children with rolandic epilepsy, also known as “childhood epilepsy with centrotemporal spikes” in the revised International League Against Epilepsy classification [1]. Rolandic epilepsy is the most common type of childhood epilepsy (17–25% in the 5–14-year age range), [2, 3] with prevalence of 1.1 in 1000 children [3]. Onset is typically around 7 years of age and seizures cease by adolescence. The seizures are focal, often nocturnal, affecting the face, arm and sometimes whole body. Seizures in rolandic epilepsy can usually be controlled with monotherapy antiepileptic medication. It is often associated with speech, attention, language, literacy, and motor impairments but not associated impairments such as autism or intellectual disability [4–6].

The primary outcome in trials evaluating interventions for epilepsy is typically freedom from seizures, or significant reduction in frequency, duration and intensity of seizures at a defined time point after commencing treatment. However, balance is required between seizure control and potential side effects of antiepileptic drugs and the impact these have on children. Health-related quality of life has also become a focus for research, with the social and psychological consequences of seizures and children’s perspectives becoming more valued [7]. At least 11 questionnaires have been developed to assess quality of life in children with epilepsy, each with a slightly different focus [8]. It is salutary to recognise that epilepsy-specific quality of life is not solely determined by seizures, but more influenced by the child’s learning, mental health and social support [9, 10]. The variety of outcomes assessed and different ways outcomes are measured, as well as the variable quality of outcome measures, are a barrier to integrating findings from studies, hence we need to ascertain a core set of more important outcomes that matter to families [11].

There is increasing recognition that identifying a core set of outcomes to be evaluated and reported in all trials of interventions for particular conditions will improve the usefulness of research, [12, 13] and avoid waste of research effort [14]. The development of a core outcome set (COS) should, at the least, include the views of patients, carers and health professionals [13]. The COS may also be useful for other types of research, clinical audit and as routinely collected health services clinical data. A COS specifies both what aspects of health are to be assessed and how the measurement will be determined.

Currently there is no established core outcome set for children and young people with epilepsy. The Core Outcome Measures in Effectiveness Trials (COMET) Initiative database includes a study focused on West syndrome [15] and a reference to a core outcome sets for adults with epilepsy [16]. Children are included in the Common Data Elements recommended for epilepsy research by the National Institute of Neurological Disorders and Stroke [17]. This recommends a comprehensive list of items across various domains but not a core set of outcomes, and children and parents were not consulted in that process.

Cochrane reviewers recommend focusing on longer-term outcomes and psychosocial, quality of life and health economic outcomes [18]. National Institute for Health and Care Excellence (NICE) guidance recommends seizure freedom as the primary outcome, and seizure reduction, quality of life and cognitive functioning as secondary outcomes [19]. Scottish guidance additionally highlights aspects of academic attainment, anxiety and depression [20]. Adverse effects from treatment that have been monitored include drug toxicity, daytime sleepiness, behavioural problems, vertigo, headaches, nausea/vomiting, diarrhoea, tremor, fatigue and rashes. The International League Against Epilepsy has also published general guidance on outcome measurement in clinical trials [21, 22].

Many regulatory agencies now mandate incorporating patient-reported outcome measures in evaluative research [23, 24]. Decisions on the adoption or reimbursement of new health technologies require evidence of cost-effectiveness derived from preference-based measures [25]. However, there remains a lack of consensus about the optimal way to estimate health utilities, given that adults, children and young people perceive and value health differently. Important considerations in the context of children and young people, therefore, include the role of proxy reporting of health utilities and the appropriateness and validity of direct and indirect methods of preference elicitation [26].

The aim of this work is to identify a core set of outcome measures for rolandic epilepsy. This study will address: (1) what outcomes to measure and (2) how to measure those outcomes. These decisions will be considered in consultation with young people with epilepsy, parents, charities and health, education and psychology professionals. The involvement of children and parents is crucial, to ensure the outcomes measured are meaningful to inform decisions about treatment, and to ensure that assessment tools are appropriate and acceptable.

This study is part of a programme of research aiming to improve broad health-related quality of life for children with epilepsy by evaluating different treatment strategies. The findings of this study will inform decisions about outcomes to be measured in a trial evaluating interventions for rolandic epilepsy scheduled to begin recruitment in 2019. Our work is motivated by the necessity to change the agenda from a seizure-centred medical model towards broader child and family priorities, and to focus scarce resources on the most important outcomes we identify [27].

## Methods

**Fig. 1:**
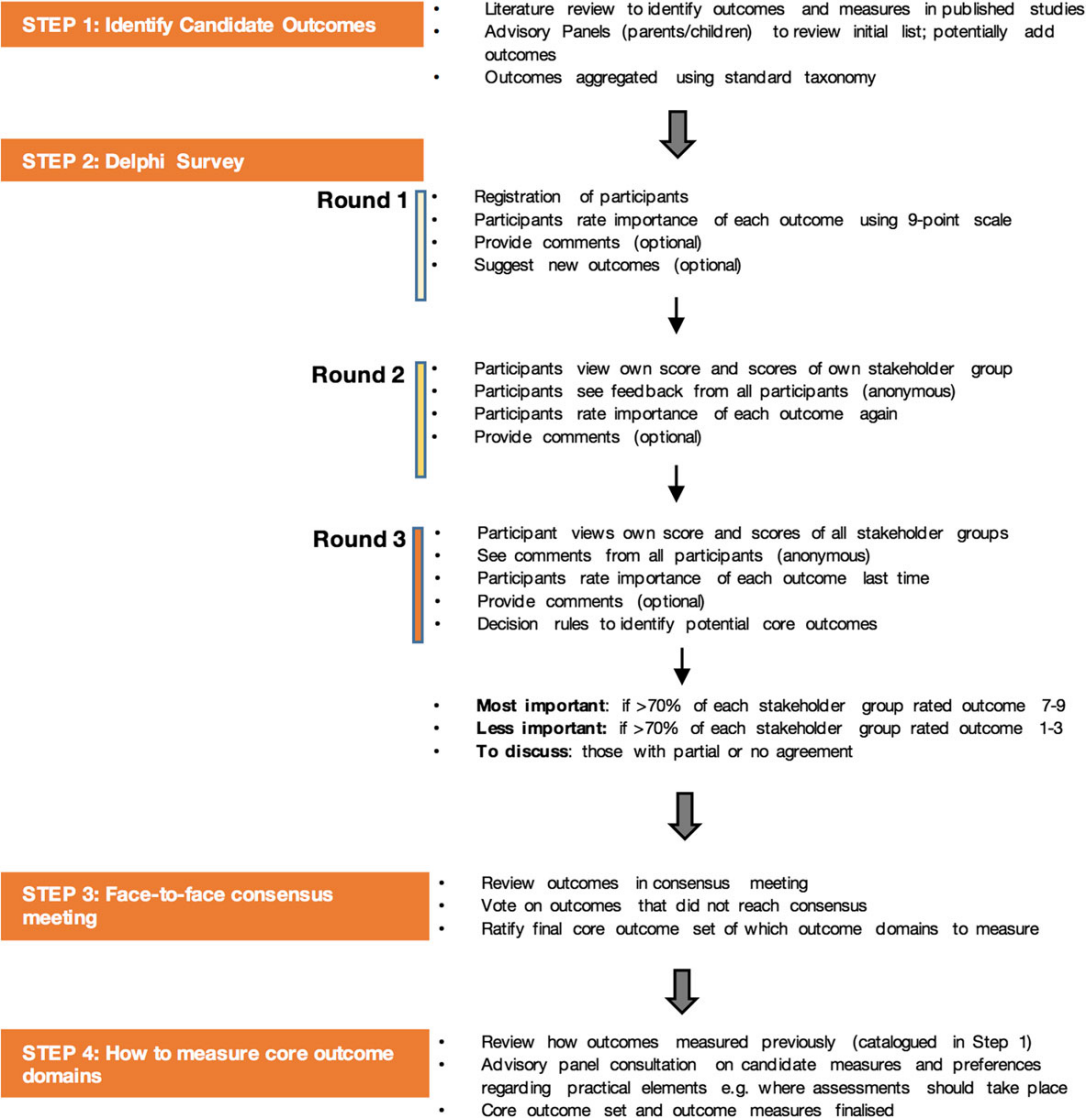
Key steps in the process

The study will be registered with the COMET Initiative and follows its procedures (Fig. 1). We will seek a proportionate ethics approval through the National Health Service (NHS) Health Research Authority and informed consent from participants.

### Scope

The study focuses on children of school age (5–16 years old) with rolandic epilepsy. The work will be conducted in partnership with families, charities and health professionals in the UK. The scope includes outcomes of any intervention where the aim is to improve the health of children with epilepsy and is not limited to drugs. Our primary objective is to propose a COS for evaluative trials but may also inform decisions about outcomes to be measured in audits such as Epilepsy12 [28, 29] and routinely collected services data.

### Public involvement in the research

We value involving children, young people and parents as meaningful partners in carrying out this research [30]. Two parents of children with epilepsy who also have experience of epilepsy themselves were co-applicants in securing funding. Two advisory panels will be convened, one with children and young people with epilepsy and one with parents of children with epilepsy. The advisory panels will be consulted at all stages where key influential decisions are required. Members of the panels will be recruited through liaising with relevant UK charities (Young Epilepsy, Epilepsy Action, Epilepsy Research, Epilepsy Society and Cerebra).

### Phase 1: identifying candidate outcomes

The following methods are proposed as a proportionate and expedient approach for creating a list of candidate outcome domains. We expect duplication where the same outcomes are measured in numerous studies. We will not re-record duplicated outcomes unless measurement was made using a different method. The search will cease when further searching does not reveal new outcomes.

#### Search strategy

Candidate outcome domains will be identified from systematic reviews and primary research studies retrieved by electronic searches run on the Cochrane Database of Systematic Reviews, CENTRAL, PsycInfo, CINAHL, Medline and EMBASE (via Ovidsp) and the World Health Organisation International Clinical Trials Registry Platform for ongoing trials. The Turning Research Into Practice (TRIP) database will be searched for national statutory guidance documents. The electronic searches will be carried out by an information specialist (MR) and results catalogued in Endnote reference manager. The strategy will use both controlled headings (e.g. medical subject headings (MeSH)) and free text. Terms will be grouped by concepts and combined accordingly. The search strategies will be recorded and reported. Search results will be managed in reference management software and the dates of searches recorded.

#### Types of studies

Eligible publications will be systematic reviews of trials, clinical trials and observational studies focusing on children with epilepsy, and national statutory guidance. We will consider qualitative research or mixed methods studies that report children’s or their parents’ preferences for outcomes. We will compile a list of epilepsy-specific and generic patient-reported outcome measures used with children with epilepsy. Only English-language papers will be included.

#### Types of interventions

We will include publications on all types of interventions that aim to improve the health of children with epilepsy, including drug trials, social interventions and psychological therapies.

#### Types of participants

Although principally focusing on rolandic epilepsy, we will include publications of studies that included study populations comprising up to 50% children (5–16 years old) with other types of epilepsy. Studies focusing predominantly on adults as participants will be excluded; to be eligible, study populations must comprise at least 80% children.

#### Exclusion criteria

Studies that include children with epilepsy but where the focus was on any associated impairments (e.g. cerebral palsy, autism) will be excluded.

#### Deciding eligibility

The study researcher will screen titles and abstracts and select references. If there are any doubts about eligibility the final decision will be made in consultation with other members of the research team. We do not propose two people screening as the additional resources are not justified by the risk of missing outcome domains, as we expect considerable duplication. A “Preferred reporting items for systematic reviews and meta-analyses” (PRISMA) flow chart will be used to record each stage of the study selection process [31].

#### Data extraction

The study researcher will record each outcome domain measured in an eligible paper and the instrument/method for ascertaining measurement. We will note participant characteristics, type of intervention where appropriate, time to measurement of outcome after the intervention was started and any other salient details relating to the practicalities of measurement. Data extracted will be checked for accuracy by a second reviewer, for quality assurance.

#### Coding, aggregation and classification of outcomes

Outcome domains will be listed in a spreadsheet and coded with reference to the taxonomy proposed by the COMET Initiative (forthcoming). The Wilson and Cleary conceptual model of patient outcomes will be used initially to sort outcomes between biological and physiological factors, symptoms, functioning, general health perceptions and overall quality of life [32].

Members of the advisory panels will meet to review and discuss the initial list of outcomes and may make suggestions for additional outcomes, aggregating outcomes or changing terminology and definitions of each outcome to make them accessible in terms of language.

### Phase 2: rating the importance of outcome domains

We will carry out a Delphi survey following recommended practices in the development of core outcome sets.

#### Stakeholders

We will engage relevant UK charities to advertise the opportunity to families of children with rolandic epilepsy to participate in the survey (e.g. Young Epilepsy, Epilepsy Action, Epilepsy Research, Epilepsy Society, Roald Dahl’s Marvellous Children’s Charity, epilepsy charities in Scotland, Wales and Northern Ireland, Cerebra and the umbrella groups Joint Epilepsy Council and Neurological Alliance). Health, education and neuropsychology professionals (e.g. paediatricians, paediatric neurologists, epilepsy nurses, clinical and educational psychologists) will be invited to participate through professional societies and special interest groups (e.g. British Paediatric Neurology Association, Royal College of Nursing, Epilepsy Nurses Association, Royal College of Paediatrics and Child Health, British Academy of Childhood Disability and British Association for Community Child Health, neurological and neuropsychology special interest groups of the British Psychological Society, Medicines for Children Research Network, NHS regional Paediatric Epilepsy Networks and Cochrane Epilepsy). We will seek to engage with general practitioners, health services managers, commissioners and policymakers through the Paediatric Neurosciences Clinical Reference Group. We will also use social network sites to link with health professionals through online communities (e.g. WeCYPnurses and #ExpofCare).

#### Survey administration

Potential participants will be invited to register through an online system or by contacting the study team and document their consent to take part. For families we will record the age of the child, the region of the UK where they live and ethnicity. We will ask whether the child wants to take part in the survey themselves independently from their parents. Whilst we suspect this task may be cognitively difficult for some children, particularly younger children, we want to be as inclusive as possible and will consider ways to modify the task for children in consultation with our young people’s advisory panel. For professionals we will record speciality (paediatrician, neurologist, nurse etc.) and the region of the UK in which they work. There is no formal sample-size calculation appropriate for this type of study; neither are there are recommendations for the number of participants to include in a Delphi survey. We will continue to recruit until we are satisfied that we have diversity of participants from all stakeholder groups. Although we target around 50–100 participants from each stakeholder group, we propose 20–30 to be the minimum. The number of each type of professional is also a function of what is realistically possible; for instance, there are fewer than 100 paediatric neurologists in total in the UK. Registration will imply consent to participate in the surveys. Each participant will have a unique identifier so we are able monitor their responses, identify their stakeholder group, and send reminder messages to non-responders. The survey will be administered using the COMET Initiative DelphiManager software.

#### Delphi survey

The first round of the survey will show the list of outcomes identified in the review and endorsed by the advisory panels. Participants will be asked to rate the importance of measuring each outcome domain in research using a 9-point scale, where response options 1–3 will indicate “less important”, options scored 4–6 as “important but not critical”, and 7–9 as “crucially important”. An optional free-text box will enable participants to explain responses or raise issues. There will also be an option to respond “not sure”/“don’t know”. In the first round participants will be offered the opportunity to identify any additional aspects of health they feel are important but have not been included. These will be considered for inclusion prior to round 2 based on whether or not they are already conceptually represented by an existing outcome domain.

The ratings of importance will be aggregated separately for families and professionals. Provided there are sufficient numbers of responses, we may explore differences between children and parents and between sub-categories of health professionals. We expect instances where one stakeholder group but not the other indicates domains more or less important.

Rounds 2 and 3 of the Delphi survey will present participants with the results from the previous round. All participants will see aggregated results for each separate stakeholder group and their own personal ratings from the previous round. Providing feedback from all stakeholder groups separately is proposed to enhance consensus between the groups [33]. They will be asked to reflect on the feedback and rate again the importance of each outcome in research with free-text boxes to indicate the rationale for their decisions and any changes.

In judging consensus, we will examine survey responses by families and health professionals separately. Over three rounds we expect to identify categories of (1) most important “core” outcomes agreed by most stakeholders (>70% in each stakeholder group rated 7–9), (2) less important outcomes agreed by most stakeholders (> 70% in each stakeholder group rated 1–3), and (3) those where there is partial or no agreement across stakeholder groups.

#### Final meeting

A face-to-face meeting will be convened to ratify a final core set of outcomes. The final round of the Delphi survey participants will be invited to volunteer to take part in this meeting. We will select at random from those who volunteer to seek a mix of young people, parents and professionals; around 20 participants in total will be ideal. Facilitated small group and plenary discussions and potentially card-sorting techniques (such as modified Q-sorting) [34] will be used to seek consensus on those outcome domains where there is disagreement.

### Phase 3: how to measure the key outcomes

Having decided which core outcome domains to measure, the next stage is to identify ways to assess the outcomes with reference to published guidance [35]. We will search for candidate outcome measures and appraise peer-reviewed evidence of measurement properties of those measures with reference to criteria for acceptable measurement properties [36]. The guideline for selecting outcome measures recommends a comprehensive systematic review be conducted for each outcome domain [35]. Given one of the purposes of this study is to inform outcomes to be measured in a subsequent trial starting in 2019, systematic reviews of all outcome domains is not feasible. Therefore, in this study, we will carry out a proportionate review of measures for each outcome with reference to methods for assessing outcome recorded in our initial review of trials, and focus particularly on reviewing patient-reported outcome measures.

For patient-reported outcome measures, including preference-based measures, we will review which questionnaires include content that matches more closely the outcome domains selected for the COS. Candidate measurement tools will be discussed with our advisory panels to identify measures judged to be most acceptable to children and parents.

#### Search strategy

We will conduct a search for evaluations of the measurement properties of each candidate patient-reported outcome measure assessing epilepsy-specific quality of life. A previous review has identified the main likely contenders [8]. Electronic searches will be run using the names and acronyms of the measures combined with terms for children, and terms for measurement properties (e.g. validity, reliability, responsiveness). Search strategies and dates of searches will be recorded. Search results will be managed in reference management software.

#### Types of studies

Eligible publications will be evaluations of measurement properties. We will only include evaluations of English-language versions of the questionnaires [37]. Only English-language papers are included.

#### Types of participants

The participants are children with epilepsy ages 5–16 years.

#### Exclusion criteria

Studies that include children with epilepsy but where the focus is more on any associated impairments (e.g. cerebral palsy, autism) will be excluded.

#### Deciding eligibility

The study researcher will screen titles and abstracts and select references. If there are any doubts about eligibility the final decision will be made in consultation with other members of the research team. A PRISMA flow chart will be used to record each stage of the study selection process [26].

#### Data extraction

The study researcher will extract evidence of measurement properties from included studies and record a structured appraisal of the methodological quality of the study [38].

#### Synthesis

Evidence of measurement properties for each patient-reported outcome measure will be considered individually and a summary appraisal rating given based on methodological quality, consistency of exceeding recommended criteria and replication of performance, especially by groups other than the developers.

Finally, our advisory panels will also be consulted about (1) preferences for where assessments should take place ideally, (2) how frequently and how long each assessment should last, (3) ways to make assessment more engaging and (4) preferences for duration of follow up in the trial.

### Dissemination

The full report and academic publication will report with reference to the Core Outcome Set–Standards for Reporting (COS-STAR) statement and checklist [39]. We will also produce a plain-language summary for non-academic audiences and an easy-read version for children. We will also seek to tell people the results and implications via social media using video and audio files.

## Discussion

The proposed methods will produce a COS for evaluative research of interventions aiming to improve the health of children with rolandic epilepsy. The findings will also inform decisions about outcomes to be measured in audits such as Epilepsy12 [28] and also routinely collected service outcomes. The findings may also be generalised to other types of common childhood epilepsy. We debated whether the scope of this work should be broader to include other epilepsy syndromes. The aim of our decision to focus principally on rolandic epilepsy as an exemplar is to avoid preferences for outcomes that might be affected by including children who have associated conditions such as autism or cerebral palsy.

The scope of our work is primarily in the UK but may have broader international relevance. Studies such as this, which are dependent on engaging families and health professionals, are always influenced by the views of the self-selecting sample of people who choose to participate. Thus the COS we propose will benefit from wider consultation once published, and potentially replication to refine either which health domains to measure or how to measure them. Of course new ways to measure the outcomes may also appear in time. Therefore, whilst the findings will inform a subsequent randomised controlled trial as part of this programme of research, and help improve the consistency of epilepsy research in the short term, it will benefit from being reviewed in future.

A number of decisions have to be made when designing a study to develop a COS [13]. Although we are not undertaking new qualitative research in this study, there can be a place for qualitative research in the early stages to seek and capture first hand which aspect of health the patients and carers value and how they articulate these potential outcomes [40]. We are reviewing published qualitative research on children’s and parents’ preferences that may elicit preferences for important outcome domains; however, we are not undertaking new qualitative research in this study. We invested substantially in advisory panels to enable children and young people with epilepsy and parents to be involved as partners in the process and ensure we use accessible language. The reviews of previous research have also been determined pragmatically rather than designed to be exhaustive. Selection of measures for each domain in the core outcome set will be based on a limited review, comprehensive systematic reviews for each domain could be planned in future research. Despite the limitations we consider the methodology proportionate to produce the core outcome set expediently, and representing value for the funding agency [41].

Selection of which stakeholders to include was another decision we debated. In addition to patients, carers and health professionals, some developers of core outcome sets have engaged pharmaceutical and/or other industry partners. We decided not to engage commercial partners; whilst drug treatment is a mainstay for seizure control it is not the only intervention within our scope. Also, the ethos of our programme of research is to encourage a broader patient-relevant and family-focused outcome framework to guide health services [27]. Our definition of health professionals includes managers and commissioners as well as clinical staff. We will report the extent to which we were able to engage professionals with less specific interest in epilepsy and any methods that were successful. Some core outcome set developers advocate the engagement of clinical trialists as a separate stakeholder group. However our team, which includes clinical trialists, decided this was not a vital group compared to families and clinicians.

Details of the core outcome set we recommend will be disseminated in a variety of ways and thereby the core outcome set will be made available for researchers to consider for implementation in evaluative research and other applications.

### Trial status

This study is not a trial. We have not begun recruitment for the Delphi study at the time of submission; recruitment will begin in January 2018 subject to ethics approvals being confirmed.

## Abbreviations

COMET: Core Outcome Measures in Effectiveness Trials
COS-STAR: Core Outcome Set – Standards for Reporting Statement and checklist
MeSH: Medical subject headings
NHS: National Health Service
NICE: National Institute for Health and Care Excellence
PRISMA: Preferred reporting items for systematic reviews and meta-analyses
